# The chronic kidney disease perception scale (CKDPS): development and construct validation

**DOI:** 10.1186/s12882-020-02028-9

**Published:** 2020-10-07

**Authors:** Haryati Anuar, Shamsul Azhar Shah, Abdul Halim Abdul Gafor, Mohd Ihsani Mahmood

**Affiliations:** 1 Department of Community Health, Faculty of Medicine, Universiti Kebangsaan Malaysia, Jalan Yaakob Latiff, Bandar Tun Razak, 56000 Cheras, Kuala Lumpur, Malaysia; 2 Faculty of Pharmaceutical Sciences, UCSI University, Kuala Lumpur, Malaysia; 3 Disease Control Division, Ministry of Health Malaysia, Putrajaya, Malaysia

**Keywords:** Chronic kidney disease perception scale, Factor analysis, Construct validity

## Abstract

**Background:**

Chronic kidney disease has become a major health problem around the world. It displays no symptoms until the later stages. Therefore, its early detection is crucial, and a suitable intervention is necessary to halt its development. The aim of this study was to develop and validate a recently formulated Chronic Kidney Disease Perception Scale (CKDPS) for diabetic patients based on Social Psychology, and their perceptions based on the Health Belief Model (HBM).

**Methods:**

The newly developed CKDPS instrument was tested on 300 patients with diabetes mellitus in a cross-sectional study. The number of domains, model-fit index, construct validity, and internal consistency of this instrument were determined using exploratory (EFA) and confirmatory factor analysis (CFA).

**Results:**

The EFA yielded nine domains: illness identity, timeline motivation, medical practice and co-operation for Social Psychology, and perceived benefit, perceived barriers, perceived susceptibility, perceived severity, and perceived cue to action for HBM. Four items with low factor loading were removed. CFA yielded the following fit indices for Social Psychology: the goodness of fit index (GFI) = 0.889, comparative fit index (CFI) = 0.934, root mean square error of approximation (RMSEA) = 0.053, normed chi-square (NC) = 1.831; and the following for HBM: GFI = 0.834, CFI = 0.957, RMSEA = 0.053, NC = 1.830. Values of Cronbach’s α ranged between 0.760 and 0.909.

**Conclusions:**

The CKDPS includes 61 questions across nine domains, divided under two categories of Social Psychology and HBM. It is also a valid and reliable tool for measuring diabetic patients’ perception of CKD prevention that can be used in larger studies.

## Background

Chronic kidney disease (CKD) has become a major health problem affecting different aspects of human life around the world. It is often called a silent killer because it displays no symptoms until the later stages. Therefore, early detection of the disease is crucial, and suitable intervention is necessary to halt or delay its development [1, 2].

CKD, also known as chronic kidney failure, is defined as a gradual kidney function loss over at least 3 months, posing a major health problem. In developing countries, it has skyrocketed into a growing epidemic, with over 20 million individuals having some degree of kidney failure. The rising elderly population and aggregate issues of obesity, hypertension, diabetes, and cardiovascular diseases facilitate its growth. There are about 26 million American adults reporting CKD and millions more are at high risk. Early detection can help prevent kidney failure [3]. CKD ranks 11th among Malaysia’s top 50 causes for deaths and 97th worldwide [4].

The increased incidences of End-Stage Renal Disease (ESRD) in developing economies among the elderly and diabetics is a major concern because it increases the cost of treatment for a therapeutic method, which already consumes a large share of the health care budget [5]. Malaysia’s task is to stop the rising tide of ESRD. The World Health Organization (WHO) estimated that over 30% of CKD cases could be prevented by monitoring the risk factors [3]. To reduce suffering and promote early detection with an accurate diagnosis, effective treatment is needed. Therefore, there must be adequate tests and services where the positive outcomes outweigh the risk of harm from the test.

### Significance

Thus far, CKD remains one of Malaysia’s most chronic diseases, and is reported to increase dramatically over the years. Although the government has made many efforts to prevent CKD, the death rate are a public concern. Factors affecting the public’s attitude towards diabetic patients need to be examined extensively so that community involvement in prevention programs is effective.

Those at a higher risk of developing CKD were not screened, making them particularly vulnerable to a late diagnosis [6]. Proactive interpretation has led to people with symptoms being more likely to receive treatment for any health problem and to frequently use medical services. Studies have indicated that specific barriers to CKD screening include the perception of it being unpleasant, complicated, and uncomfortable [7–9].

Nonetheless, there is no accurate or effective instrument to assess the CKD screening test’s expectations of a diabetes mellitus patient. This is an instrument report developed in accordance with an established methodological process.

### Objective of the study

The main purpose of this study was to construct a questionnaire to evaluate CKD perception among diabetic patients, and test its reliability and validity based on structural equation modeling (SEM) and confirmatory factor analysis (CFA) compliance.

## Methods

### Participants and data acquisition

Data were collected at Primer Clinics operated by the Canselor Tuanku Muhriz Hospital, Kuala Lumpur District, from April to June 2017. Through cross-sectional sampling, 300 respondents with diabetes mellitus, aged between 31 and 87 years, were recruited. An interview was conducted to recruit those respondents who met the inclusion criteria. The interview session was held in the waiting area for those respondents who had completed the informed consent form, after which they were promptly informed of the study’s objective. Respondents meeting the following parameters were recruited: (1) not diagnosed with CKD, (2) diagnosed with diabetic mellitus, and (3) not mentally ill.

The CKDPS was read to participants who could not read well. They could also ask the main researcher for clarity as to whether their response was vague or complicated. To ensure there were no missing answers, their replies were thoroughly checked [6]. Structural Equation Modeling (SEM) is a very powerful multivariate test method that describe a network of relationships between variables. SEM is important to look at integrated multivariate relationships and the use of CFA in measurement error correction. The standard SEM graphic vocabulary are: (1)  latent variables, factors or constructs, (2)  observed variables, measures, indicators manifest variables, (3)  direct effects, (4)  correlation or covariance [10].

### Instrument validation

This stage aimed to define the initially established CKDPS domains, consisting of 65 items to fill the knowledge gap in CKD among diabetes mellitus patients. The questionnaire items were developed using socio-psychology and HBM to assess validity and internal consistency. During the Exploratory Factor Analysis (EFA) stage factors loading less than 0.4 were removed and the Confirmatory Factor Analysis (CFA) stage was discriminator and convergence-based, with average variance extracted (AVE) and composite reliability (CR) values. The sample size in this pilot test was based on 1 item of question ratio to count with 5 respondents, thus, the total number of sufficient respondents required for pilot this test was 300. Lastly the pilot study was conducted before proceeding with the real original sample size.

All items were developed to represent the properties of nine socio-psychology and HBM domains on CKD perception based on DeVells’ (2003) scale creation protocol [11]:

1. *Domain development*: Based on a thorough review of quantitative and qualitative literature on Social Psychology and the perception of CKD, this instrument was built after reviewing other models, such as the Common-Sense Model (CSM) and the Protection Motivation Model (PMM). The HBM was selected based on its advantages over other models and being a framework-related scale.
2. *Generating an item pool*: The primary pool of items were developed based on an in-depth analysis of HBM-guided component articles and a limited number of available model theories. The initial pool comprised 61-items: seven items measuring illness identity, six items measuring timeline motivation, eight items measuring medical practice, and seven items measuring social psychology cooperation. The HBM design included seven items each to measure benefit, barrier, cue to action and severity, and nine items to measure susceptibility.
3. *Determining the format*: The CKDPS had a Likert-scale format. Items are rated on a Likert 10-point scale ranging from 1 = strongly disagree to 10 = strongly agree. The category of perception was determined by selecting a score value representing 25% of the highest value as acceptable, while 75% of the lowest value was considered weak. The scores were in the range of 163–489. Value at the top of 25% and lowest at 75% on the graph score distribution is the determining point. This selection has the correct percentage at the higher value because of the various socio-psychology and health belief model of perceptions of CKD prevention are readily available. However, the central parameter is not affected by the value generated from any defective data.
4. *The initial sample of items reviewed by experts*: The finalized questionnaire was completed under Lynn’s (1986) guidance [12]. Five experts were asked to review the questionnaire for content validity. Construct-oriented questions were generated through the collaboration of trained healthcare professionals, based on the instrument’s theoretical and conceptual basis. Therefore, the experts assessed the accuracy of items and their relationship with the domain, eventually deciding if these items should stay in the pool. A verified questionnaire for face validation was pre-tested by 30 health care workers. Minor changes were made prior to the validation trial, following a change after recommendations for new items were suggested. The instrument, originally in English, was translated into Malay by a qualified bilingual translator who checked both the versions for language accuracy [13].

### Statistical analysis

Two domains with 65-items were analyzed using the IBM SPSS statistical software, and CFA was performed using the IBM SPSS Amos version 25. The data obtained were analyzed using the statistical program Monte Carlo PCA, to systematically compare its own values greater than 1 in SPSS with those provided by the parallel analysis. Constructs omitted all loading factors below 0.4. The EFA was then extended to varimax and oblimin rotation. Both the Kaiser-Meyer-Olkin (KMO) sample adequacy index and Bartlett’s Sphericity Test determined item checking. Reliability analysis was performed on all domains, collected before and after removing items at this stage.

Data was sent for further CFA. Path analysis showed the discriminator and convergence features of the average variance extracted (AVE) and composite reliability (CR) value. Correlation values were estimated using maximum probability estimates to assess whether the model fitted the covariance matrix of the approved data set as recommended, i.e., a comparative fit index (CFI) of 0.9 [14], goodness of fit index (GFI) of > 0.9 [15], normed fit index (NFI) of 0.9 [16], and the root mean square error of approximation (RMSEA) of 0.05 to 0.1 [17] and 5.0 [18]. Items remained stable after the retention of EFA and CFA [19].

## Results

### Respondents

A total of 300 questionnaires were sent, answered and returned for analysis. Respondents were aged 31–87 years with an average of 62.24 years and a standard deviation of ±9.3 years. Female respondents comprised 53.7% of the sample, while male respondents comprised 46.3%. In terms of work, many were pensioners (75.3%) and most of whom were 52% Malays.

### Exploratory factor analysis

A principal component analysis (PCA) of the CKDPS was carried out, to investigate the Social Psychology domain dataset with a KMO value of 0.789 and Bartlett’s sphericity test at *p* <  0.001 (see Table 1). The findings were appropriate, suggesting the utility and suitability of this data for factor analysis. For the HBM domain, PCA showed a KMO value of 0.806 and a Bartlett sphericity test significant at *p* <  0.001 (see Table 1).

**Table 1:** KMO and Bartlett’s test result

KMO and Bartlett’s test	
**Socio-psychology**	
KMO measure of sampling adequacy	0.789
Bartlett’s test of sphericity	
Approximate chi-square	3321.556
Degree of freedom	378
Significance	< 0.001
**Health belief model**	
KMO measure of sampling adequacy	0.806
Bartlett’s test of sphericity	
Approximate chi-square	4847.848
Degree of freedom	666
Significance	< 0.001

The exploratory testing method aimed at reducing the questionnaire items to a reasonable number while retaining the reliability and subjacent structures of the scale. Items with a factor loading of 0.4 or less were removed from item selection. Of the 28 items, only 2 were removed; the rest represented seven factors. The seven constructs demonstrated satisfactory reliability as shown by Cronbach’s 0.7 alpha value. The items representing the six other constructs were considered the main building sub-constructions. The result was interpreted as predicting four models. Another PCA was performed, where many items from the seven combined constructs combined provided a four-construction solution with items mounted on the same constructs as before. Table 2 shows an average reliability rate of 0.86, with high reliability from 0.8 to 0.9. Therefore, factor analysis was necessary. Only 2 of the 37 items were eliminated, with the remaining items reflecting five constructs. All items with a factor loading of 0.4 or below were excluded. Therefore, the outcome was interpreted as five constructs as expected by the model, known as perceived severity, perceived susceptibility, perceived benefits, perceived barriers, and cue to action. Of these, “perceived barrier” had three subdomains; “perceived susceptibility” had three subdomains; and the other domains were consistent with the expected theoretical model. Another PCA was developed. Several objects from the five constructs were combined, with items loaded onto the same constructs as before. Table 3 reveals that the overall scale reliability was 0.87 with strong subscale reliability, α = 0.8–0.9.

**Table 2:** Factor analysis for 28 items in socio-psychology for CKDPS

Items	Mean (SD)	Fear	Timeline and motivation	Medical practice	Co-operation	ITC	Alpha
C1	8.52 (0.807)	0.637				0.443	0.749
C2	8.59 (0.695)	0.752				0.494	
C3	8.61 (0.748)	0.738				0.494	
C4	8.75 (0.729)	0.741				0.455	
C5	8.58 (0.701)	0.643				0.421	
C6	8.59 (0.733)	0.651				0.395	
C7	8,81 (0.741)	0.489				0.549	
C8	3.69 (0.989)		0.516			0.185	0.590
C9	4.02 (0.989)		0.710			0.468	
C10	4.20 (0.977)		0.615			0.299	
C11	3.99 (0.885)		0.640			0.361	
C12	3.82 (0.953)		0.612			0.343	
C13	4.44 (0.892)		0.612			0.318	
C14	3.37 (2.297)			0.851		0.670	0.631
C15	2.76 (2.028)			0.867		0.507	
C16	3.13 (2.266)			0.907		0.662	
C17	4.95 (2.149)			0.624		0.458	
C18	6.22 (2.095)	0.498				0.374	
C19	7.12 (1.624)	0.455				0.258	
C20	4.01 (2.913)					0.096	
C21	7.33 (2.402)				0.757	−0.157	
C22	7.85 (1.275)				0.759	0.675	0.886
C23	8.07 (1.291)				0.840	0.683	
C24	8.01 (1.393)				0.738	0.775	
C25	7.94 (1.631)				0.799	0.647	
C26	8.18 (1.264)				0.820	0.692	
C27	8.09 (1.266)				0.697	0.713	
C28	8.14 (1.189)					0.584

**Table 3:** Exploratory factor analysis for 37 items in health belief model for CKDPS

Items	Mean (SD)	Perceived benefit	Perceived barrier	Perceived susceptibility	Perceived severity	Perceived cue to action	ITC	Alpha
D1	6.75 (1.708)	0.757					0.713	0.918
D2	6.54 (1.517)	0.816					0.757	
D3	7.05 (1.782)	0.806					0.801	
D4	6.76 (1.544)	0.872					0.847	
D5	6.85 (1.503)	0.850					0.843	
D6	6.96 (1.749)	0.309					0.755	
D7	7.59 (1.425)	0.477					0.527	
D8	3.14 (2.465)			0.663			0.089	0.460
D9	2.65 (2.115)			0.737			0.346	
D10	2.59 (1.849)			0.552			0.412	
D11	3.31 (2.328)			0.422			0.371	
D12	8.10 (2.230)			0.530			−0.038	
D13	4.25 (1.961)				0.704		0.277	
D14	3.90 (1.985)				0.710		0.137	
D15	5.25 (2.174)					0.593	0.401	0.605
D16	6.19 (2.144)					0.698	0.404	
D17	5.39 (2.289)					0.699	0.404	
D18	8.45 (1.528)		0.423				0.306	
D19	5.49 (2.108)					0.552	0.332	
D20	4.10 (2.115)					0.530	0.376	
D21	4.40 (2.293)			0.420			−0.025	
D22	5.72 (1.831)			0.329			0.233	
D23	7.67 (1.752)					0.449	0.243	
D24	7.91 (1.179)		0.549				0.492	0.835
D25	8.36 (1.105)		0.619				0.452	
D26	7.53 (1.550)		0.464				0.508	
D27	7.83 (1.379)		0.600				0.534	
D28	8.20 (1.213)		0.720				0.713	
D29	8.38 (1.191)		0.815				0.718	
D30	8.45 (1.128)		0.840				0.741	
D31	7.22 (1.783)				0.552		0.121	0.537
D32	6.32 (2.520)				0.640		0.083	
D33	3.70 (2.835)			0.560			0.300	
D34	3.31 (2.221)			0.599			0.481	
D35	3.22 (2.199)			0.492			0.454	
D36	5.95 (2.029)			0.427			0.233	
D37	7.52 (1.750)		0.566				0.253	

Out of 65 items, 4 were removed, leaving 61 items with 18 constructs. The 18-factor structure not only demonstrated an acceptable level of reliability shown by a Cronbach’s alpha value of 0.7, but also the items representing nine Social Psychology and HBM contructs. Meanwhile another PCA was performed. Most of the 18 constructs produced 9 construct components with values above 1.0 and a total reliability range between 0.789 and 0.806 and a good reliability range of 0.8 to 0.9 [6].

### Factor-label analysis

This section discusses and describes the theoretical and practical basis of each aspect of the CKD Screening Perception Scale constructs using Social Psychology and HBM [6].

#### Construct A

Component 1, illness identity fear, comprises seven items with a credible reliability value of 0.866. These items were extracted as the first component and represented more perceptions than the other components. An example of this component is, “I need to reinforce my lifestyle modifications” (Item C23).

#### Construct B

Component 2, timeline and motivation comprises nine items with a reliability value of 0.909. This component is a combination of two and seven items from two different components, and concerns the positive efforts of individuals regarding compliance, physicians, and knowledge of CKD perceptions. Therefore, this component is labeled “timeline and motivation.” Examples of statements for this component are: “By giving knowledge to my family members I can increase their positive CKD perception” (Item C7) and “I will only take medicine if I feel sick instead of taking it daily” (Item C18). Both examples indicate why these components have been labeled as they have.

#### Construct C

Component 3, medical practice, comprises five items with a sub-component value of 0.760. Most of these items relate to the consequences for respondents and reducing risks or complications among diabetes mellitus patients that end up with CKD. Each of the questions showed good perceptions of preventing CKD by monitoring the health status. An example statement of this component is, “CKD can lead to death” (Item C14).

#### Construct D

The last component, cooperation, comprises six items from one original component. The reliability value is 0.817. This component reflects alarm and concern for health conditions in CKD prevention. Some examples are “I am sure that CKD will affect my quality of life” (Item C10), and “I am afraid of CKD” (Item C11). Therefore, this component has been labeled thus.

#### Construct PB

Perceived benefit (PB) comprises six items with a reliability of 0.875; most of the items relate to the efficacy of the intervention considered in reducing the risk of harm. Responses to the CKD prevention scale were labeled PB. For example, “Control of dietary meal can prevent the development of CKD” (Item D6).

#### Construct PBR

Perceived Barriers (PBR) comprises nine items with a subscale reliability of 0.900. Three sub-constructions were merged into PBR, since most of the subjects appeared to be related to the barriers of CKD prevention among diabetic patients. Therefore, it was labeled PBR. Examples include “CKD can be fatal” (Item D12) and “I would consider compliance toward treatment of CKD” (Item D15).

#### Construct PSS

Perceived Susceptibility (PSS) comprises nine items with a subscale reliability of 0.909. This construction consisted of two sub-domains and merged four items each from a different construct, as most of the items appeared to be related to the critical clinical or social consequences of disease diagnosis. It was therefore labeled PSS. An example is, “I don’t have time to go to my monthly check-up” (Item D18).

#### Construct PS

Perceived severity (PS) comprises five items with a subscale reliability of 0.833. Originally, this construct was similar to the concept of developing a disorder, and hence was combined into a single construct. This is related to factors of perceived severity (PS), for example, “the cost of treatment is high” (Item D25).

#### Construct CA

Cues for action (CA) comprises six items with a subscale reliability of 0.875. The purpose of this concept is the activation of preventive action and hence, was labeled accordingly. Some examples include “I feel healthy” (Item D35) and “I am not at risk of CKD” (Item D34).

### Confirmatory factor analysis

Using AMOS version 25 [18], the initial path analysis in CFA for social psychology was performed through an exploratory factor structure. It is shown that the four domains and 26 questions obtained from the EFA had poor model fit values, with CMIN / DF = 2.550 (*n* = 300), *p* < 0.001, GFI = 0.835, CFI = 0.826, RMSEA = 0.072 (*p*_Close_ < 0.001) and SRMR = 0.138. For a good model fit value, objects with a factor loading below 0.6 and a Modification Index (MI) above 10, should have been omitted. However, given the importance of the questions based on the patients’ perspective using social psychology, they were retained in the model. The results improved significantly, with CMIN / DF = 1.831 (*n* = 300), *p* < 0.001, GFI = 0.889, CFI = 0.934, RMSEA = 0.053 (*p*_Close_ = 0.263), and SRMR = 0.053, showing a close alignment of the final model with the data. The results significantly improved.

**Fig. 1:**
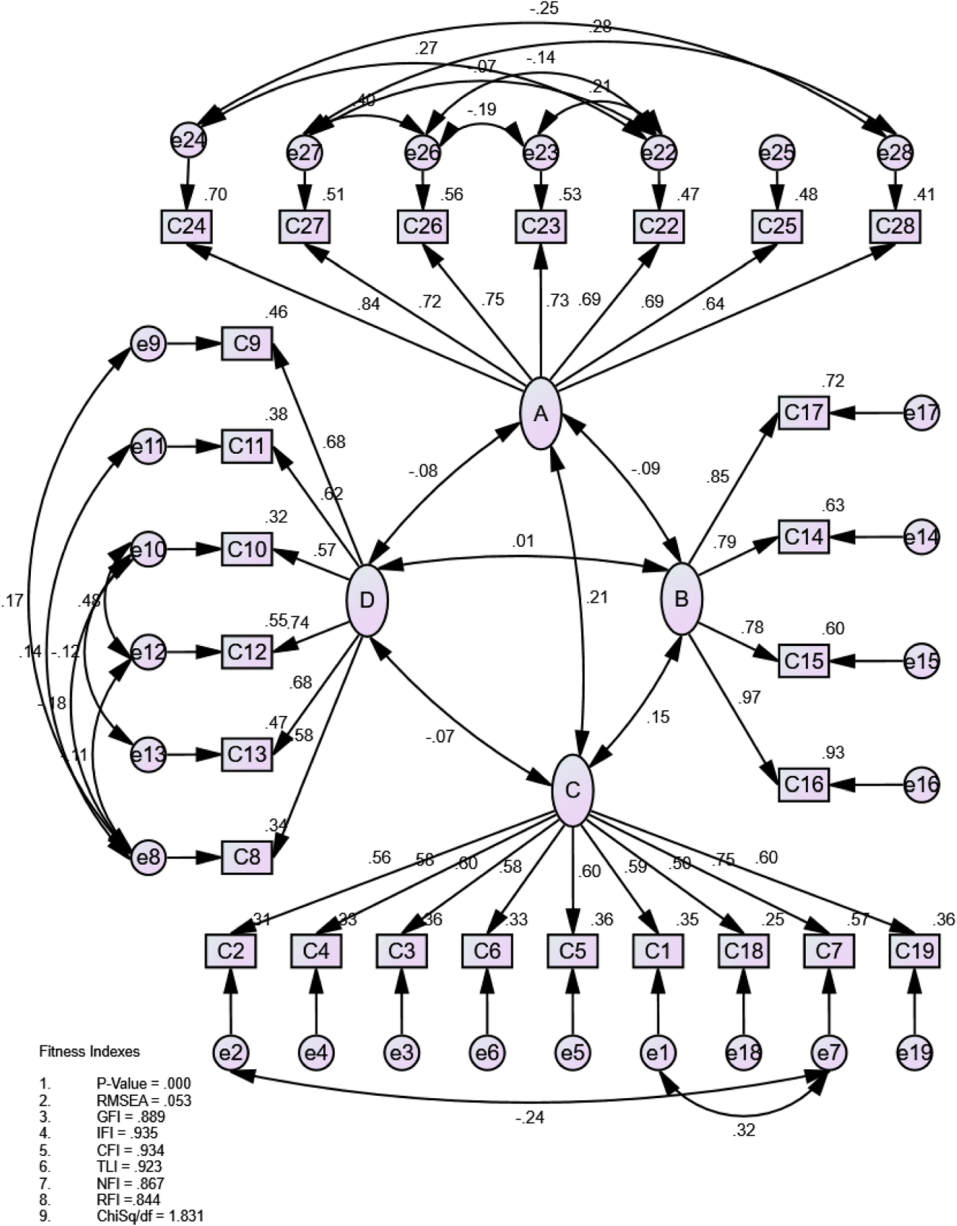
Confirmatory factor analysis of the four factors and 26-items of social psychology

Figure 1 summarizes the final route review, with 26 items relating to the four domains reflecting the constructions measured. The loading factor (represented by a single-headed arrow connecting the rectangles to ellipses) for retained items in their respective domains, ranged from 0.594 to 0.966. The double-headed arrow indicates the value of the four domain correlations. Domains of the same constructs were strongly correlated while the domains of different constructs were slightly correlated. The A domain, for example, was moderately interconnected to the C domain of construction experience (*r* = 0.66), but weakly interrelated with the B and D domains (*r* = 0.15) (Fig. 1).

**Fig. 2:**
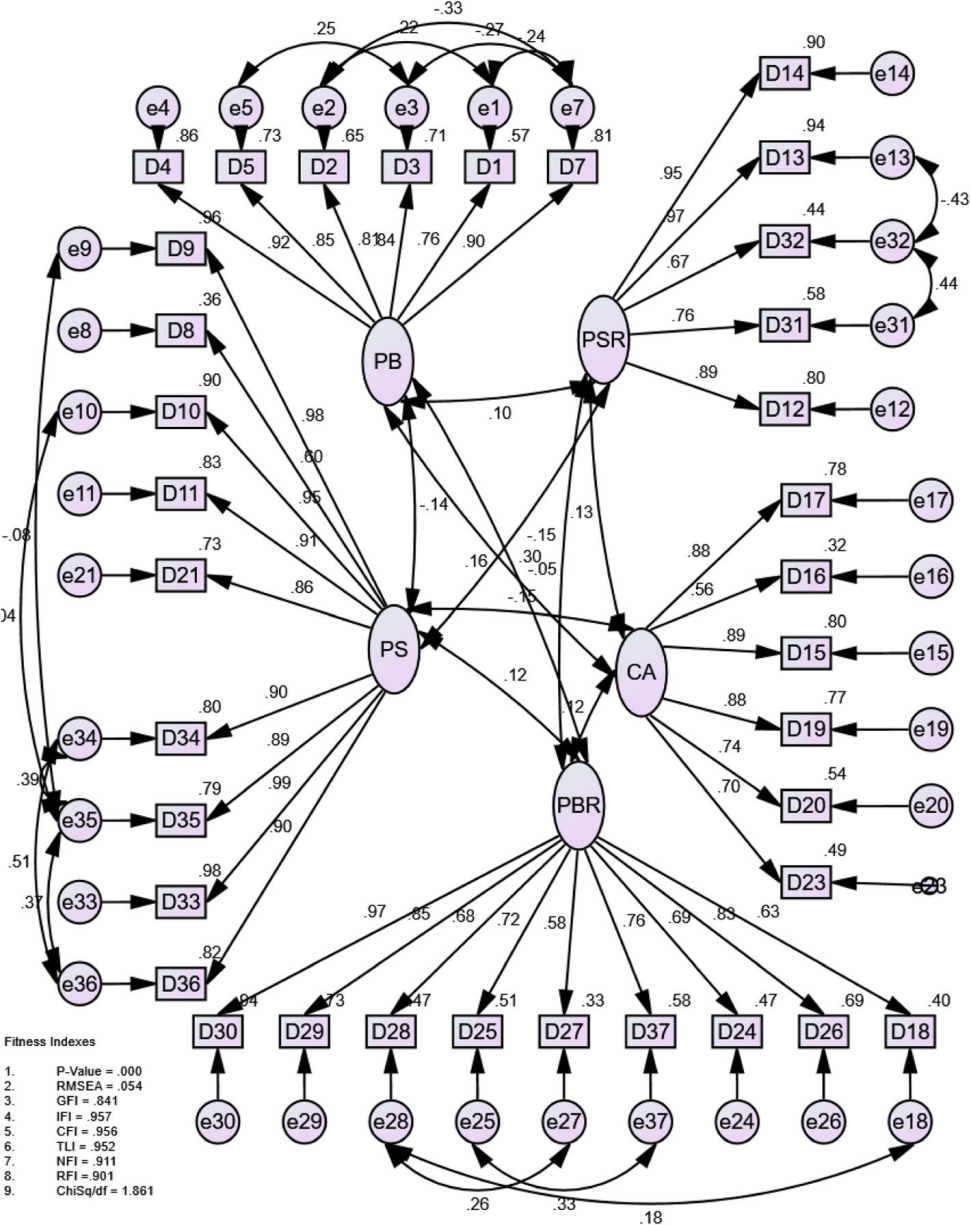
Confirmatory factor analysis of the five-factor 35-item health belief model

Figure 2 presents a CFA path analysis, which indicates standardized interaction estimates (arrow figures) between the five domains (ellipses), the 35 domains (rectangles) and residuals (circles). The CONFIRM data pack was well-balanced and the same structure was confirmed based on five domains with 35-items produced from the EXPLORE dataset. While the outcome was declared fair, it was a relatively poor match based on the goodness of fit statistics of the 37-items listed in the exploratory data set, namely CMIN / DF = 2.581 (*n* = 300), *p* < 0.001, GFI = 0.774, CFI = 0.916, RMSEA = 0.001 and SRMR = 0.204, respectively. Items below 0.6 and an MI above 10, should have been removed for the improvement of the fitness of the model. Nevertheless, they remained a paradigm because of the significance of these issues in understanding the patient experience. Consequently, the remaining modified items in the index were linked to the covariance line by more than 10. With CMIN / DF = 1.839 (*n* = 300), *p* < 0.001, GFI 0.834, CFI 0.957, RMSEA 0.053 (*p*_Close_ = 0.183) and SRMR < 0.957, the findings had significantly improved, suggesting that the final models were suitable for testing.

The final path description is shown in Fig. 2 and retains 35-items in relation to the five domains describing the measured structures. The retained items in their respective fields were loaded between 0.563 and 0.9888 (represented by a single-headed flew lining rectangles with ellipses). Arrows with double-headed links to the domain suggest similarities between the five domains. As shown in Tables 4 and 5, composite reliability and average variance show the internal consistency of all parameters. Analysis of the confirmatory factor of structural equation modeling was helpful [6].

**Table 4:** CFA results with factor loading of each item towards their respective domain (Socio-psychology) and the measurement index for construct validity and internal consistency

Domain	Item	Factor loading	Average variance extracted	Composite reliability	Cronbach’s alpha
Illness identity fear	C24	0.840	0.667	0.885	0.886
	C27	0.716			
	C26	0.751			
	C23	0.731			
	C22	0.689			
	C25	0.692			
	C26	0.643			
Timeline and motivation	C17	0.846	0.735	0.911	0.909
	C14	0.794			
	C15	0.776			
	C16	0.966			
Medical practice	C19	0.597	0.575	0.839	0.760
	C7	0.752			
	C18	0.597			
	C1	0.594			
	C5	0.596			
	C6	0.575			
	C3	0.600			
	C4	0.577			
	C2	0.560			
Cooperation	C8	0.579	0.568	0.810	0.817
	C13	0.685			
	C12	0.741			
	C10	0.566			
	C11	0.615			
	C9	0.676

**Table 5:** CFA results with factor loading of each item towards their respective domain (Health Belief Model) and the measurement index for construct validity and internal consistency

Domain	Item	Factor loading	AVE	CR	Cronbach alpha
Perceived benefit	D4	0.925	0.732	0.950	0.875
	D5	0.854			
	D2	0.809			
	D3	0.843			
	D1	0.756			
	D7	0.900			
Perceived barrier	D30	0.970	0.570	0.921	0.900
	D29	0.853			
	D28	0.683			
	D25	0.716			
	D27	0.579			
	D37	0.762			
	D24	0.689			
	D26	0.833			
	D18	0.629			
Perceived susceptibility	D9	0.981	0.787	0.973	0.909
	D8	0.603			
	D34	0.896			
	D33	0.989			
	D10	0.948			
	D35	0.890			
	D36	0.904			
	D11	0.913			
	D21	0.857			
	D22	0.832			
Perceived severity	D14	0.951	0.733	0.931	0.833
	D13	0.970			
	D32	0.666			
	D31	0.761			
	D12	0.892			
Cues to action	D17	0.882	0.616	0.904	0.857
	D16	0.564			
	D15	0.892			
	D19	0.878			
	D20	0.736			
	D23	0.703			

## Discussion

Standard measurements of validity and reliability were performed to yield appropriate constructs. Therefore, this study developed and evaluated Social Psychology and HBM as tools among diabetic patients regarding their perception of CKD prevention. The final version comprises 61-items with acceptable reliability and relevance for potential use, based on the statistical indices of the SEM. The EFA is used to make all items a common factor based on the linear pattern of the load factor. The CFA complements the approach by affirming and ensuring that the extracted elements and their respective items are inclusive and have convergent validity (construct validity). The findings of this study indicate that the Malay variant of the CKDPS has sufficient psychometric properties to assess the perception of diabetic mellitus patients with CKD.

The EFA is used to factor all items into common factors based on the pattern-linearity of the loading factor, and the CFA complements this method by confirming and ensuring the discriminatory and convergent validity (constructive validity) of the extracted factors and of their respective items. The findings of this study indicate that the Malay variant of the CKDPS has sufficient psychometric properties to assess the perception of diabetic mellitus patients to CKD.

Heir et al. [19] stated that the retained items must have a rotated loading factor of at least 0.4 (i.e., > + 0.4 or < −0.4). In other words, factorability is an important item. Items with loadings less than 0.4 were eliminated after proof from additional criteria was considered and the item was shown to be invalid [19]. The EFA had enough observations to ensure the stability, and the original 65-items were reduced to 61 to ensure that it yields usable results.

Absolute fit indices are considered because this model was not tested previously [20]. The SEM’s preliminary results, based on the exploratory 65-items, showed poor fit for the model due to its complexity, including all factors [21]; therefore, some changes were made to the model to fit the data better. Both low-factor load items were removed. The model was then revised, and the SEM was re-run. The changes included reducing some of the model’s limitations, including removing items with wider indices of transition, rather than concentrating on the main factors suggested a priori [19]. Consequently, all indices are now appropriate, and the model appears to fit the data well. That is, the data collected in this study appears to support this concept.

Overall, the revised scale’s validity and reliability were checked. The capacity of both, the experts and focus groups, to respond to these has improved content validity. Depending on these findings, some susceptibility items were removed, and some barrier items were added. The validity of the construct was evaluated using an empirical element both exploratory and confirmatory, as they represented different conceptual frameworks for validity evaluation. All three scales were unidirectional in both EFA and CFA. The susceptibility scale, even with three items, maintained very strong correlations among individual items, as found in explorative and confirmative factor analyses.

CFA is a tool for the identification of variables loaded on the relevant factors. The scale is focused on Social Psychology and HBM theory, and this approach will determine several factors and their interrelationships. Therefore, the factors on which the items are mounted can be calculated. Furthermore, path analysis is one of the components of SEM that does not presume error calculation for each variable [16]. Factor analytics can be described by specific factors by variable-only factor scores [22]. Measuring the factor values and taking them into the route analysis will mitigate the impact of measuring the error. Thankfully, this is the simulation of structural equations, which provides various benefits in CFA [21].

The AVE and CR values were determined to assess the converging/discriminating validity and reliability of each domain and are described in Table 4. The AVE values ranged from 0.56 to 0.76 and were all above the cut-off value of 0.5, suggesting that all four domains had sufficient convergent validity. The finding showed that (i) all domains had strong discriminatory validity, and (ii) all domains had strong discriminatory validity. Cronbach’s α of all domains ranged from 0.76–0.90 and was equivalent to Cronbach’s α in their respective values, suggesting that all domains had good internal consistency (Table 4).

For completing the CKDPS, fair internal consistency was observed as reliability exceeded the minimum acceptable values. Consequently, less random error than anticipated was observed, with good internal consistency due to the misinterpretation of fewer items than the respondents predicted [6].

The AVE and CR values were calculated to determine the convergent/discriminating validity and reliability of each domain and are presented in Table 5. The AVE ranged from 0.57 to 0.79 and were all above the cut-off value of 0.5, suggesting that all five domains had sufficient convergent validity. This finding shows that all domains had a strong biased validity. Cronbach’s α of all domains ranged from 0.83–0.91, and their values were comparable to each other, suggesting that all domains had good internal consistency (Table 5).

### Research strengths and weaknesses

The strength of this study is the large number of participants in the validation procedure. The sample size was based on 1 item of question ratio to count with 5 respondents, thus, the total number of respondents required for pilot test was 300. Cronbach’s alpha values were included in the reliability and internal consistency test. Even though the coefficient values were high, the items in the questionnaire were considered more consistent in the variables measure, with a Cronbach’s alpha value of 0.65 and above, was acceptable as accurate and consistent [23].

The advantage of this research is that it is the first validation study to assess CKD interpretation in Malaysia using factor analysis based on a combination of Social Psychology and HBM scales. This research has been performed in the study population (diabetic patients) by means of a technique such as the one developed for comprehensive study results and can be extended to other responsive CKD populations (obesity, cardiovascular disease and hypertension). The high 99% response rate minimizes the chance of non-response. Second, the accurate bilingual interpretation has helped to reduce the possibility of an intelligence bias by making Malaysian respondents from many different ethnic backgrounds and national language proficiencies understandable.

The review also has several limitations, in addition to its strengths. First there is a lack of a test-retest evaluation of the instrument’s reliability. It is therefore recommended that this section be included in future studies. In the meantime, the validation process is a cross-sectional analysis and there are drawbacks to the design of the study itself, as it cannot be based on performance. Analyzed data were collected only once from a population or from a representative sample subset. Memory can change over time, and participants ‘memory of the study period can only be a snapshot. In fact, the pool of very similar respondents can be scanned, but it can be hard to find a particular domain or attribute.

## Conclusion

Results show that this scale is statistically valid and reliable. A newly developed CKDPS measurement of the CKD perception test and latent variables are available. In a larger study, it could be used to test the perception based on Social Psychology and HBM. Therefore, CKDPS is a valid and reliable scale for diabetes mellitus patients and is now available.

## Data Availability

The data sets generated and analysed during the current study are available from the corresponding author on reasonable request.

## Abbreviations

HBM: Health Belief Model
CKDPS: Chronic Kidney Disease Perception Scale
CKD: Chronic Kidney Disease
SEM: Structural Equation Model
PCA: Principal Component Analysis
CFA: Confirmatory Factor Analysis
EFA: Exploratory Factor Analysis
ESRD: End Stage Renal Disease
CSM: Common Sense Model
PMT: Protection Motivation Theory
KMO: Kaiser-Meyer-Olkin
CFI: Comparative Fit Index
GFI: Goodness of Fit Index
NFI: Normed Fit Index
CMIN/DF: Chi-Square Value and Degree of Freedom
RMSEA: Root Mean Square Error of Approximation
MI: Modification Indices
AVE: Average Variance Extracted
CR: Composite Reliability
Construct A: Illness Identity Fear
Construct B: Timeline and Motivation
Construct C: Medical Practise
Construct D: Co-operation
Construct PB: Perceived Benefit
Construct PBR: Perceived Barrier
Construct PSS: Perceived Susceptibility
Construct PS: Perceived Severity
Construct CA: Perceived Cue to Action
WHO: World Health Organization
